# Switch to tenecteplase for intravenous thrombolysis in stroke patients: experience from a German high-volume stroke center

**DOI:** 10.1186/s42466-025-00388-x

**Published:** 2025-05-05

**Authors:** Alexander Sekita, Gabriela Siedler, Jochen A. Sembill, Manuel Schmidt, Ludwig Singer, Bernd Kallmuenzer, Lena Mers, Anna Bogdanova, Stefan Schwab, Stefan T. Gerner

**Affiliations:** 1 Department of Neurology, University Hospital Erlangen, Schwabachanlage 6, 91054 Erlangen, Germany; 2 Department of Neuroradiology, University Hospital Erlangen, Schwabachanlage 6, 91054 Erlangen, Germany

**Keywords:** Ischemic stroke, Intravenous thrombolysis, Tenecteplase, Alteplase, Procedure times, Delayed time window, Safety

## Abstract

**Background:**

Tenecteplase (TNK) offers promising efficacy and safety data for intravenous thrombolysis (IVT) in acute ischemic stroke (AIS) and pharmacological advantages over alteplase (rt-PA), justifying its gradual adoption as primary thrombolytic agent. At our tertiary care center, we transitioned from rt-PA to TNK, providing valuable real-world insights into this process, including its use beyond the 4.5-hour time window.

**Methods:**

We retrospectively analyzed our stroke registry to compare clinical and procedural data from AIS patients treated with rt-PA (up to 6 months before transition) and those treated with TNK (up to 6 months after transition, starting June 2024). Primary endpoints included treatment metrics, such as door-to-needle (DTN), door-to-imaging (DTI), imaging-to-needle (ITN), door-to-groin and door-to-recanalization times. Safety outcomes comprised rate of any intracranial hemorrhage (ICH), symptomatic ICH (sICH), parenchymatous hematoma type 2 (PH 2) and post-thrombolysis angioedema. A semiquantitative questionnaire evaluated satisfaction with TNK and changes in lysis behavior among nurses and physicians 3 months post-implementation.

**Results:**

During the twelve-month period (December 1, 2023 - November 30, 2024), 276 patients underwent IVT. Median DTN times were significantly shorter with TNK (*n* = 138) compared to rt-PA (*n* = 138) (TNK 27 min [IQR 19–39] vs. rt-PA 34 min [IQR 25–62]; *p* = 0.011). No significant differences were observed in safety outcomes, including any ICH (TNK 9% vs. rt-PA 6%; *p* = 0.30), sICH (2% vs. 1%; *p* = 0.31), PH 2 rates (1% in both groups), or angioedema (3% vs. 1%; *p* = 0.18). Staff satisfaction with TNK was high, citing advantages in preparation, administration, and time efficiency. Importantly, no changes in lysis behavior were reported following the transition.

**Conclusions:**

Transitioning to TNK in routine practice at a tertiary care center seems feasible with reduced ITN and consequently DTN times. Functional outcomes at discharge were comparable without significant difference in the rate of (s)ICH. Overall, the transition to TNK was well-received by medical staff, highlighting TNK’s practical advantages in acute stroke care.

**Trial registration:**

N.A.

**Supplementary Information:**

The online version contains supplementary material available at 10.1186/s42466-025-00388-x.

## Introduction

Acute ischemic stroke (AIS) is a leading cause of mortality and long-term disability worldwide [1]. Intravenous thrombolysis (IVT) and endovascular thrombectomy (EVT) for large vessel occlusion (LVO) are established treatments that significantly improve outcomes in eligible patients [2]. For decades, alteplase (rt-PA) has been the global standard as thrombolytic agent for IVT, administered as a bolus followed by a one-hour infusion [2]. Recently, however, tenecteplase (TNK) has emerged as an alternative thrombolytic agent, increasingly adopted in stroke centers worldwide [3].

TNK offers distinct pharmacological and practical advantages over rt-PA. These include a longer half-life, greater fibrin specificity, and stronger resistance to plasminogen activator inhibitor-1, enabling superior clot lysis [4]. Additionally, TNK’s single-bolus administration simplifies logistics, particularly in “drip-and-ship” settings, where rapid initiation of treatment facilitates timely EVT transfer for patients with LVO [4]. Clinical trials have demonstrated TNK’s non-inferiority to rt-PA in functional outcomes and safety measures, such as symptomatic intracerebral hemorrhage (sICH) [5–11]. Furthermore, a recent meta-analysis suggested that TNK might be superior to rt-PA in achieving excellent functional outcome and reduced disability at 3 months [12].

In February 2024, TNK received regulatory approval in Germany as an alternative fibrinolytic for AIS [13]. Despite updated guidelines endorsing TNK use, its adoption remains limited due to concerns about its use in off-label scenarios, such as administration beyond the 4.5-hour time window or in cases with “soft” contraindications [14–18]. Additionally, transitioning to TNK requires modifications to local treatment protocols, drug preparation, and standardized staff training, which may act as further barriers to implementation [19, 20].

Our center transitioned to TNK as the primary thrombolytic agent in June 2024. This study provides a real-world analysis of TNK implementation, including quality metrics, clinical and radiological outcomes, and its use beyond the 4.5-hour window. Given the substantial evidence of TNK’s efficacy and safety, we aimed to evaluate its practical benefits in reducing procedural times, such as door-to-needle (DTN), imaging-to-needle (ITN) and door-to-groin (DTG) times [12]. We also assessed the impact of TNK on lysis behavior among medical staff and their perspectives on its handling, effectiveness, and safety during the transition.

## Methods

### Study design, data acquisition and participants

This retrospective cohort study included AIS patients undergoing recanalizing therapy - either IVT, endovascular thrombectomy (EVT) or both - at the Department of Neurology, University Hospital Erlangen, enrolled in our local prospective IVT registry. In addition to our routinely performed simulation-based team training for IVT in the emergency room (ER), since April 2024, all ER nurses and physicians have participated in standardized training in preparation for transitioning the standard operating procedure (SOP) to TNK as the primary thrombolytic agent for acute stroke treatment. The training included preparation of TNK, current indication and contraindications. The transition was conducted in June 2024.

AIS patients treated with TNK over a 6 months-period (June to November 2024) were compared with those treated with rt-PA over the 6 months-period (December 2023 to May 2024), before the transition. Patients undergoing EVT without IVT were excluded from further analyses. Data were obtained from our institutional stroke registry, capturing baseline characteristics, including demographic information, medical history, neurological status, neuroradiological findings, treatment time metrics, and in-hospital outcomes.

### Survey

Three months after TNK implementation, a semiquantitative questionnaire was distributed via mail to ER nurses and physicians. The survey assessed satisfaction with distinct treatment aspects, including efficacy, safety, preparation, dosage, application, and time expenditure compared to rt-PA. Respondents also reported their professional affiliation, clinical experience, frequency of TNK use, and any observed changes in thrombolysis practices. Overall satisfaction and the impact of the transition were evaluated on a 5-item Likert scale ranging from “much worse” (1) to “much better” (5) compared with rt-PA.

### Local stroke management

Both rt-PA (Boehringer Ingelheim, 0.9 mg/kg) and TNK (Boehringer Ingelheim, 0.25 mg/kg) were administered according to current stroke guidelines and at the treating physician´s decision [14]. While rt-PA was given as a bolus followed by a one-hour infusion (maximum 90 mg), TNK was administered as a single intravenous bolus (maximum 25 mg). Pre-treatment imaging included brain computed tomography (CT; Siemens SOMATOM x.ceed) or magnetic resonance imaging (MRI; Siemens MAGNETOM Sola Fit 1.5 Tesla). IVT decisions adhered to SOPs based on non-contrast CT within the 4.5-hour window or mismatch profiles (perfusion CT or MRI) for extended windows.

Off-label IVT in patients on oral anticoagulation (OAC) was assessed individually. For vitamin K antagonists, IVT was considered up to an international normalized ratio (INR) of 1.7. For direct oral anticoagulants (DOACs), IVT was assessed for factor-Xa-inhibitors up to an anti-Xa activity of 100 ng/ml, as well as for Dabigatran up to a level of 100 ng/ml in the Hemoclot assay. Routine follow-up imaging 24 h post-IVT or earlier (in cases of neurological worsening) evaluated hemorrhagic transformation. Reperfusion success after EVT was determined by a neuroradiologist using the modified Thrombolysis in Cerebral Infarction (mTICI) score, with scores ≥ 2b considered successful. Neurological severity was assessed by the National Institutes of Health Stroke Scale (NIHSS) at admission, 24 and 72 h and at discharge. Functional outcomes were assessed at discharge using the modified Rankin Scale (mRS) and defined as excellent (mRS 0–1) and favorable (mRS 0–2) outcome.

### Outcomes

The primary outcome was the improvement of treatment metrics, including door-to-needle (DTN), door-to-imaging (DTI), imaging-to-needle (ITN), door-to-groin (DTG), and door-to-recanalization times (DTR). The time-point of the patient arriving at the CT- or MRI-suite, respectively, was recorded and used for calculation of the DTI and ITN. Secondary outcomes included functional outcomes at discharge (e.g., mRS 0–1 vs. 2–6; mRS 0–2 vs. 3–6 and neurological improvement (NIHSS improvement ≥ 4 points within 72 h). Safety outcomes included incidences of any intracranial hemorrhage (ICH), symptomatic ICH (sICH) per SITS-MOST criteria, parenchymatous hematoma type 2 (PH 2) by Heidelberg Bleeding Classification, and angioedema post-thrombolysis [21, 22]. Satisfaction with TNK compared with rt-PA was assessed using a Likert scale (1 = “much worse” to 5 = “much better”).

### Statistical analysis

Statistical analyses were performed using SPSS (version 28.0, SPSS Inc., Chicago, IL/USA; www.spss.com). A two-tailed p-value < 0.05 was considered statistically significant. Categorical variables were compared using the Chi-square test, and continuous data were analyzed using the Mann-Whitney U test for non-normally distributed variables or t-tests for normally distributed data. Results are presented as means ± standard deviations (SD), medians with interquartile ranges (IQR), or absolute and relative frequencies. Subgroup analyses were performed for patients treated beyond the 4.5-hour window. Subanalyses were conducted for subgroups of interest, i.e. early versus extended time-window (≤ 4.5 h vs. > 4.5 h) and according to stroke severity (mild stroke: NIHSS 1–4, moderate stroke: NIHSS 5–15 and severe stroke: NIHSS ≥ 16).

### Standard protocol approvals, registrations, and patient consents

This study was conducted with the approval of the ethics committee. The ethics approval number 377_17 Bc was obtained for the project titled “Clinical follow-up of patients with acute stroke” on 25 May 2020.

### Data availability

The data that support the findings of this study are not publicly available due to ethical restrictions but may be available from the corresponding author upon reasonable request and with appropriate permissions.

## Results

**Fig. 1:**
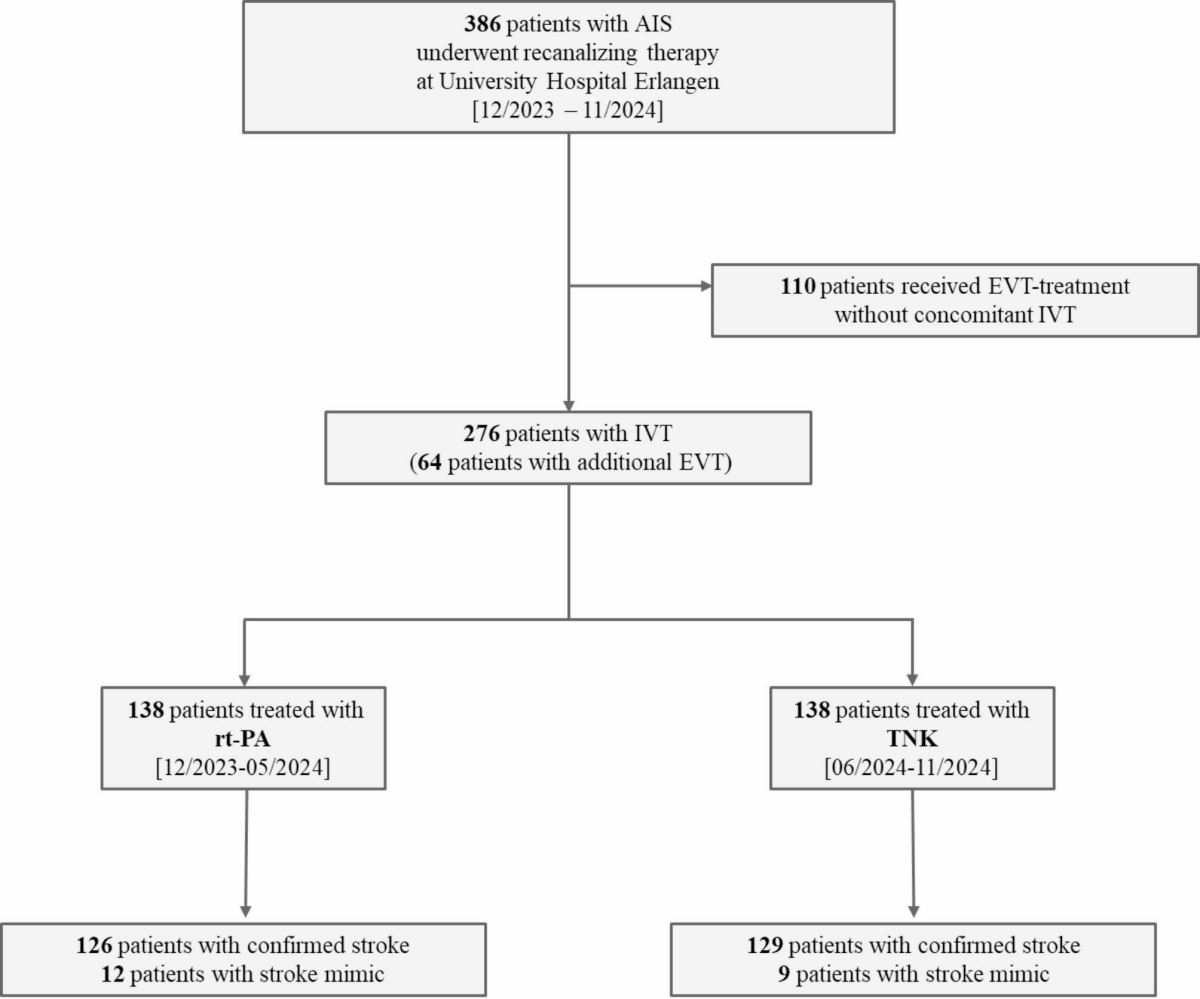
Patient Flowchart. A total of 386 AIS-patients underwent recanalizing therapy. After exclusion of patients with EVT only, 276 IVT-treated patients remained for analyses (rt-PA: *n* = 138, TNK: *n* = 138). Abbreviations: AIS indicates acute ischemic stroke; IVT, intravenous thrombolysis; EVT, endovascular treatment

Between December 1, 2023, and November 30, 2024, 386 patients presenting with suspected AIS underwent recanalizing therapy at the ER of the Department of Neurology. After excluding 110 patients treated exclusively with EVT, 276 patients treated with IVT remained for final analyses (Fig. 1). Among these, 138 patients were treated with TNK and 138 patients received rt-PA. A total of 21 patients were identified as stroke mimics during the in-hospital work-up (*n* = 12 in the TNK group and *n* = 9 in the rt-PA group, respectively; as shown in Fig. 1).

### Comparison of patient baseline characteristics

Baseline demographic and clinical characteristics, including medical history and stroke features, are summarized in Table 1. Both groups were comparable regarding demographic parameters, with a median age of 77 years (IQR 63–85) for TNK and 74 years (IQR 59–82) for rt-PA treated patients. Females represented 46% of the cohort (128/276). The median NIHSS score at presentation was similar (TNK 6 [IQR 3–11]; rt-PA 7 [IQR 4–12]). Cardiovascular comorbidities were slightly imbalanced between both groups, with a higher prevalence especially of arterial hypertension (TNK 67% vs. rt-PA 76%), vascular diseases (TNK 15% vs. rt-PA 22%) and atrial fibrillation (TNK 9% vs. rt-PA 17%) in the rt-PA group. No significant differences were observed in antithrombotic premedication, especially prior OAC use.

**Table 1:** Baseline characteristics and stroke features

AIS-patients treated with IVT (*n* = 276)	TNK (*n* = 138)	rt-PA (*n* = 138)
Age, y; median (IQR)	77 (63–85)	74 (59–82)
- Patients aged > 80 years; *n* (%)	39 (28%)	51 (37%)
Female sex; *n* (%)	60 (44%)	68 (49%)
**Prior medical history;** ***n*** **(%)**		
Premorbid mRS; median (IQR)	0 (0–2)	0 (0–3)
- Premorbid mRS 0–1	101 (73%)	89 (65%)
Heart failure	11 (8%)	19 (14%)
Hypertension	93 (67%)	105 (76%)
Diabetes mellitus Type II	27 (20%)	33 (24%)
Previous Stroke or TIA	29 (21%)	33 (24%)
Vascular diseases*	21 (15%)	30 (22%)
Atrial fibrillation	13 (9%)	23 (17%)
CHA2DS2-VASc-Score; median (IQR)	3 (2–4)	4 (2–5)
**Comedication;** ***n*** **(%)**		
OAC intake	17 (12%)	17 (12%)
Single antiplatelet therapy	44 (32%)	45 (33%)
Dual antiplatelet therapy	2 (1%)	0 (0%)
**Stroke characteristics**		
NIHSS on admission; median (IQR)	6 (3–11)	7 (4–12)
Stroke severity; *n* (%)		
NIHSS 0	9 (7%)	2 (1%)
Mild (NIHSS 1–4)	47 (34%)	47 (34%)
Moderate (NIHSS 5–15)	62 (45%)	68 (49%)
Severe (NIHSS ≥ 16)	20 (15%)	20 (15%)
Symptom-onset-to-door, min; median (IQR)**	80 (60–143)	78 (56–119)
- Early time window(≤ 4.5h); *n* (%)	99 (72%)	104 (75%)
- Late time window (> 4.5h); *n* (%)	39 (28%)	34 (25%)
- Unknown time window; *n* (%)	0 (0%)	1 (1%)
Large vessel occlusion present; *n* (%)	40 (29%)	45 (33%)
- Carotid artery; *n* (%)	6 (15%)	8 (18%)
- Carotid T; *n* (%)	2 (5%)	7 (16%)
- M1; *n* (%)	13 (33%)	15 (33%)
- M2; *n* (%)	17 (43%)	11 (24%)
- Basilar artery; *n* (%)	1 (3%)	2 (4%)
- Vertebral artery; *n* (%)	1 (3%)	2 (4%)
Endovasular thrombectomy; *n* (%)	31 (78%)	33 (73%)
**Initial imaging modality;** ***n*** **(%)**		
NCCT	4 (3%)	5 (4%)
PCT	130 (94%)	130 (94%)
MRI	1 (1%)	2 (1%)
Imaging performed externally	3 (2%)	1 (1%)
**IVT based on imaging modality;** ***n*** **(%)**		
NCCT-based	70 (51%)	72 (52%)
PCT-based	66 (48%)	64 (46%)
MRI-based	2 (1%)	2 (1%)
**Toast classification;** ***n*** **(%)**		
Microangiopathy	22 (17%)	12 (9%)
Macroangiopathy	25 (19%)	37 (28%)
Cardioembolic	40 (31%)	47 (36%)
Unknown	38 (30%)	32 (24%)
Other	4 (3%)	4 (3%)

*defined as presence of coronary artery disease, peripheral artery disease or aortic plaque

**exclusively patients with known time of symptom-onset (*n* = 166)

Abbreviations: AIS indicates acute ischemic stroke; IVT, intravenous thrombolysis; IQR, interquartile range; mRS, modified Rankin Scale; TIA, transient ischemic attack; OAC, oral anticoagulation; NIHSS, National Institutes of Health Stroke Scale; NCCT, non-contrast computed tomography; PCT, perfusion computed tomography; MRI, magnetic resonance imaging;

### Treatment and stroke characteristics

Patients presented more often within the early (≤ 4.5 h) (TNK 72% vs. rt-PA 75%) than in the extended (> 4.5 h) time window (TNK 28% vs. rt-PA 25%). Median symptom-onset-to-door times were 80 min (IQR 60–143) for TNK and 78 min (IQR 56–119) for rt-PA. LVO was identified in 29% of cases for TNK and in 33% of cases for rt-PA, with EVT performed in 64 patients overall (TNK 31/40 [78%] vs. rt-PA 33/45 [73%]). Occlusion sites were similar, predominantly in the anterior circulation, i.e. 38 out of 40 (95.0%) TNK-patients and 41 out of 45 (91.1%) rt-PA-patients. IVT was predominantly performed based on non-contrast-CT (TNK 51% vs. rt-PA 52%) or perfusion-CT (TNK 48% vs. rt-PA 46%) (Table 1).

### Procedural metrics and early outcomes

Table 2 summarizes treatment metrics and outcomes. The TNK group had a significantly shorter DTN time compared to the rt-PA group (median 27 min [IQR 19–39] vs. 34 min [IQR 25–62], *p* = 0.011). Similarly, the TNK group demonstrated a significantly shorter ITN time (median 11 min [IQR 9–16] vs. 17 min [IQR 10–31], *p* = 0.037). IVT within 30 min of admission was achieved more frequently in the TNK group than in the rt-PA group (53% vs. 40%, *p* = 0.030). Other procedural times, such as DTI, DTG, and DTR, did not differ significantly (Table 2 and Supplementary Fig. 1). Early neurological outcomes, assessed as NIHSS improvement (i.e. ≥4 points improvement during the first 72 h; TNK 36% vs. rt-PA 39%; *p* = 0.53), and rate of early recanalization prior to EVT (for LVO only) were comparable between both groups (Table 2). Recanalization rates and successful reperfusion (mTICI 2b/3) were also similar, with successful reperfusion achieved in 87% of TNK and 91% of rt-PA-treated patients (*p* = 0.63).

**Table 2:** In-hospital outcomes

	TNK (*n* = 138)	rt-PA (*n* = 138)	*P* Value
**Procedure times, min; median (IQR)**			
Door-to-needle	27 (19–39)	34 (25–62)	0.011
Door-to-imaging	15 (9–21)	17 (13–22)	0.12
Imaging-to-needle	11 (9–16)	17 (10–31)	0.037
Door-to-groin	65 (57–80)	72 (64–106)	0.17
Door-to-recanalization	97 (80–127)	111 (88–152)	0.25
Door-to needle within 30 min; *n* (%)	73 (53%)	55 (40%)	0.030
**Procedure times for evident strokes (NIHSS > 4, time window ≤ 4.5h), min; median (IQR)**			
Door-to-needle	25 (17–46)	31 (23–50)	0.018
Door-to-imaging	14 (9–17)	15 (11–20)	0.21
Imaging-to-needle	11 (7–23)	14 (8–24)	0.041
Door-to-groin	60 (55–77)	71 (64–105)	0.05
Door-to-recanalization	90 (78–104)	106 (97–148)	0.15
Door-to needle within 30 min; *n* (%)	36 (67%)	31 (48%)	0.046
**Clinical in-hospital outcomes during the first 72h**			
Early major NIHSS improvement (≥ 4 within 72h); *n* (%)	49 (36%)	54 (39%)	0.53
Early major NIHSS deterioration (≥ 4 by day 1 or 3)*; *n* (%)	16 (12%)	21 (15%)	0.38
Early recanalization pre-EVT; *n* (%)	3 (10%)	1 (3%)	0.27
Cancellation of IVT; *n* (%)	0 (0%)	2 (1%)	0.16
**Radiological outcomes**			
mTICI after EVT**; *n* (%)			0.87
mTICI 3	20 (65%)	21 (64%)	
mTICI 2c	2 (7%)	3 (9%)	
mTICI 2b	5 (16%)	6 (18%)	
mTICI 2a	1 (3%)	0 (0%)	
mTICI 0	3 (10%)	3 (9%)	
Successful reperfusion (mTICI 2b-3); *n* (%)	27 (87%)	30 (91%)	0.63

*except intubated patients

**in patients with large vessel occlusion

Abbreviations: IQR, interquartile range; NIHSS, National Institutes of Health Stroke Scale; EVT, endovascular treatment; IVT, intravenous thrombolysis; mTICI, modified thrombolysis in cerebral infarction

### Safety outcomes

Follow-up imaging revealed low rates of ICH across both groups (Table 3, and Supplementary Fig. 2). Any ICH occurred in 9% of TNK and 6% of rt-PA cases (*p* = 0.30), while sICH rates were 2% and 1%, respectively (*p* = 0.31). Parenchymal hematoma (PH2) was rare, occurring in only 1% of patients in each group. Angioedema was similarly rare (TNK 3%, rt-PA 1%, *p* = 0.18). Subgroup analyses showed no significant differences in safety outcomes across time windows, stroke severity categories or in cases of LVO or concurrent oral anticoagulation.

**Table 3:** Safety outcomes

Safety outcomes	TNK (*n* = 138)	rt-PA (*n* = 138)	*P* Value
**Overall**			
Any ICH; *n* (%)	13 (9%)	8 (6%)	0.30
Symptomatic ICH*; *n* (%)	3 (2%)	1 (1%)	0.31
Parenchymal hematoma (PH2); *n* (%)	2 (1%)	1 (1%)	0.56
Angioedema after IVT; *n* (%)	4 (3%)	1 (1%)	0.18
**Subgroups**			
- Time window ≤ 4.5h (*n* = 186)			
Any ICH in IVT within 4.5h; *n* (%)	8 (9%)	5 (5%)	0.67
sICH in IVT within 4.5h; *n* (%)	3 (3%)	1 (1%)	0.30
- Time window > 4.5h (*n* = 90)			
Any ICH in IVT > 4.5h; *n* (%)	5 (11%)	3 (7%)	0.29
sICH in IVT > 4.5h; *n* (%)	0 (0%)	0 (0%)	-
- Patients with LVO (*n* = 85)			
Any ICH in LVO; *n* (%)	4 (10%)	5 (11%)	0.63
sICH in LVO; *n* (%)	0 (0%)	1 (2%)	0.34
- Patients with minor stroke (*n* = 94)			
Any ICH in minor stroke (NIHSS 1–4); *n* (%)	2 (4%)	1 (2%)	0.50
sICH in minor stroke (NIHSS 1–4); *n* (%)	1 (2%)	0 (0%)	0.32
- Patients with moderate stroke (*n* = 130)			
Any ICH in moderate stroke (NIHSS 5–15); *n* (%)	8 (13%)	2 (3%)	0.06
sICH in moderate stroke (NIHSS 5–15); *n* (%)	2 (3%)	0 (0%)	0.14
- Patients with severe stroke (*n* = 41)			
Any ICH in severe stroke (NIHSS ≥ 16); *n* (%)	3 (15%)	5 (24%)	0.45
sICH in severe stroke (NIHSS ≥ 16); *n* (%)	0 (0%)	1 (5%)	0.32
- Patients under concurrent OAC (*n* = 34)			
Any ICH in severe stroke (NIHSS ≥ 16); *n* (%)	0 (0%)	3 (18%)	0.07
sICH in severe stroke (NIHSS ≥ 16); *n* (%)	0 (0%)	1 (6%)	0.31

Abbreviations: ICH, intracranial hemorrhage; IVT, intravenous thrombolysis; sICH, symptomatic intracranial hemorrhage; LVO, large vessel occlusion; NIHSS, National Institutes of Health Stroke Scale; OAC, oral anticoagulation

### Clinical outcomes at discharge

**Fig. 2:**
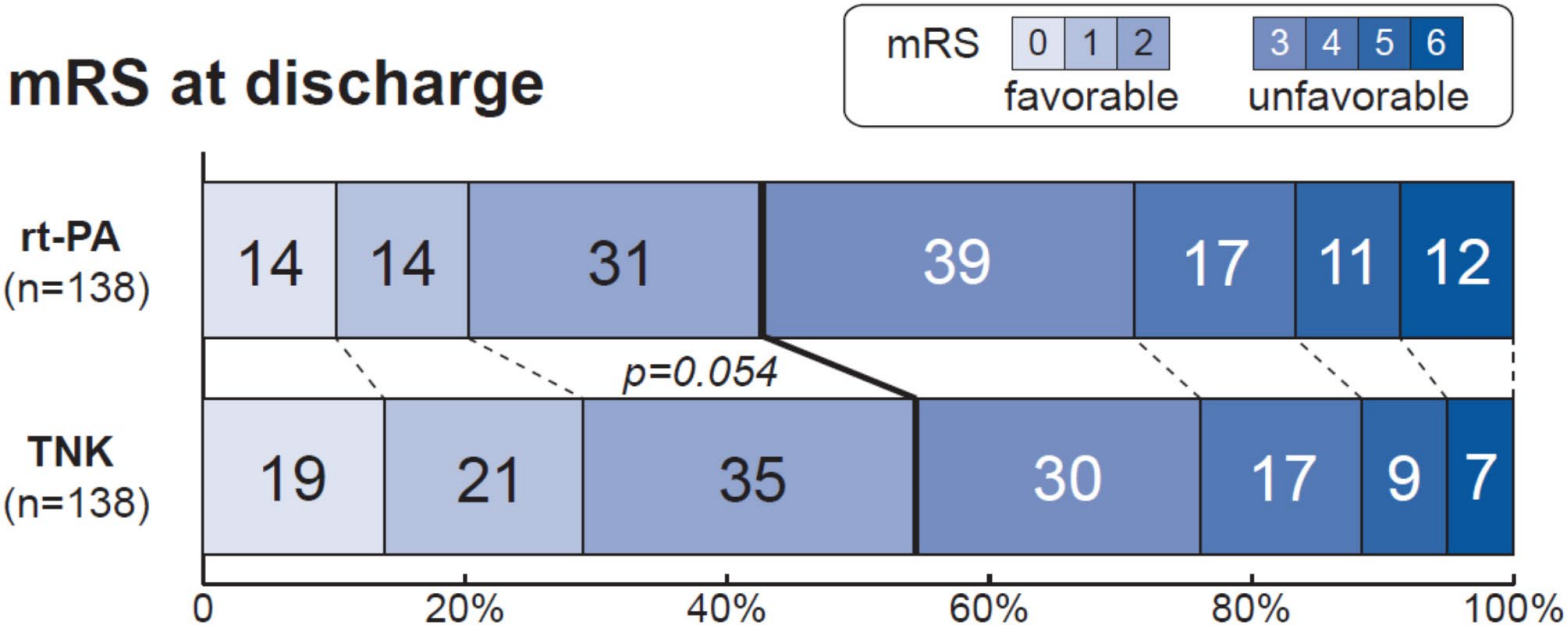
Distribution of scores on the Modified Rankin Scale at discharge. The bars show the number of participants and the scale indicates the proportion of participants in percentage. Outcome was dichotomized into favorable (i.e. mRS = 0–2) and unfavorable (i.e. mRS = 3–6). Abbreviations: mRS indicates modified Rankin Scale

The median mRS score at discharge was 2 (IQR 1–3) for TNK and 3 (IQR 2–4) for rt-PA (*p* = 0.06; Table 4). Excellent neurological recovery (mRS 0–1) was achieved in 29% of TNK patients and 20% of rt-PA patients (*p* = 0.09), while good recovery (mRS 0–2) was observed in 54% and 43 %, respectively (*p* = 0.05; Fig. 2). When considering recovery to pre-stroke mRS levels, excellent recovery rates were 43% for TNK and 37% for rt-PA (*p* = 0.33), with favorable recovery (mRS 0–2 or pre-stroke mRS) at 67% and 57% (*p* = 0.11). In-hospital mortality rates were low in both groups (TNK 5% vs. rt-PA 9%, *p* = 0.24), and the median length of hospital stay was 5 days for both groups (*p* = 0.33), with similar discharge destinations between both groups (home: TNK 64%, rt-PA 52%; rehabilitation: TNK 23%, rt-PA 26%; nursing home: TNK 6%, rt-PA 7%).

**Table 4:** Clinical outcomes at hospital discharge

	TNK (*n* = 138)	rt-PA (*n* = 138)	*P* Value
mRS score at discharge; median (IQR)	2 (1–3)	3 (2–4)	0.06
Excellent neurological recovery at discharge (mRS 0–1); *n* (%)	40 (29%)	28 (20%)	0.09
Good neurological recovery at discharge (mRS 0–2); *n* (%)	75 (54%)	59 (43%)	0.05
Excellent neurological recovery at discharge (mRS 0–1) or back to pre-mRS; *n* (%)	59 (43%)	51 (37%)	0.33
Favorable neurological recovery at discharge (mRS 0–2) or back to pre-mRS; n (%)	92 (67%)	79 (57%)	0.11
NIHSS score at discharge, median (IQR)	2 (0–4)	1 (0–5)	0.30
mRS 5–6 at discharge; *n* (%)	16 (12%)	23 (17%)	0.23
In-hospital mortality; *n* (%)	7 (5%)	12 (9%)	0.24
Length of hospital stay, d; median (IQR)	5 (3–9)	5 (3–11)	0.33
**Discharge destination;** ***n*** **(%)**			
Death	7 (5%)	12 (9%)	0.26
Rehabilitation	31 (23%)	36 (26%)	
Nursing home	8 (6%)	9 (7%)	
Home	88 (64%)	72 (52%)	
Other clinic	4 (3%)	9 (7%)	
Stroke mimics; *n* (%)	12 (9%)	9 (7%)	0.73

Abbreviations: mRS, modified Rankin Scale; IQR, interquartile range; pre-mRS, pre-stroke modified Rankin Score; NIHSS, National Institutes of Health Stroke Scale

### Healthcare provider satisfaction

**Fig. 3:**
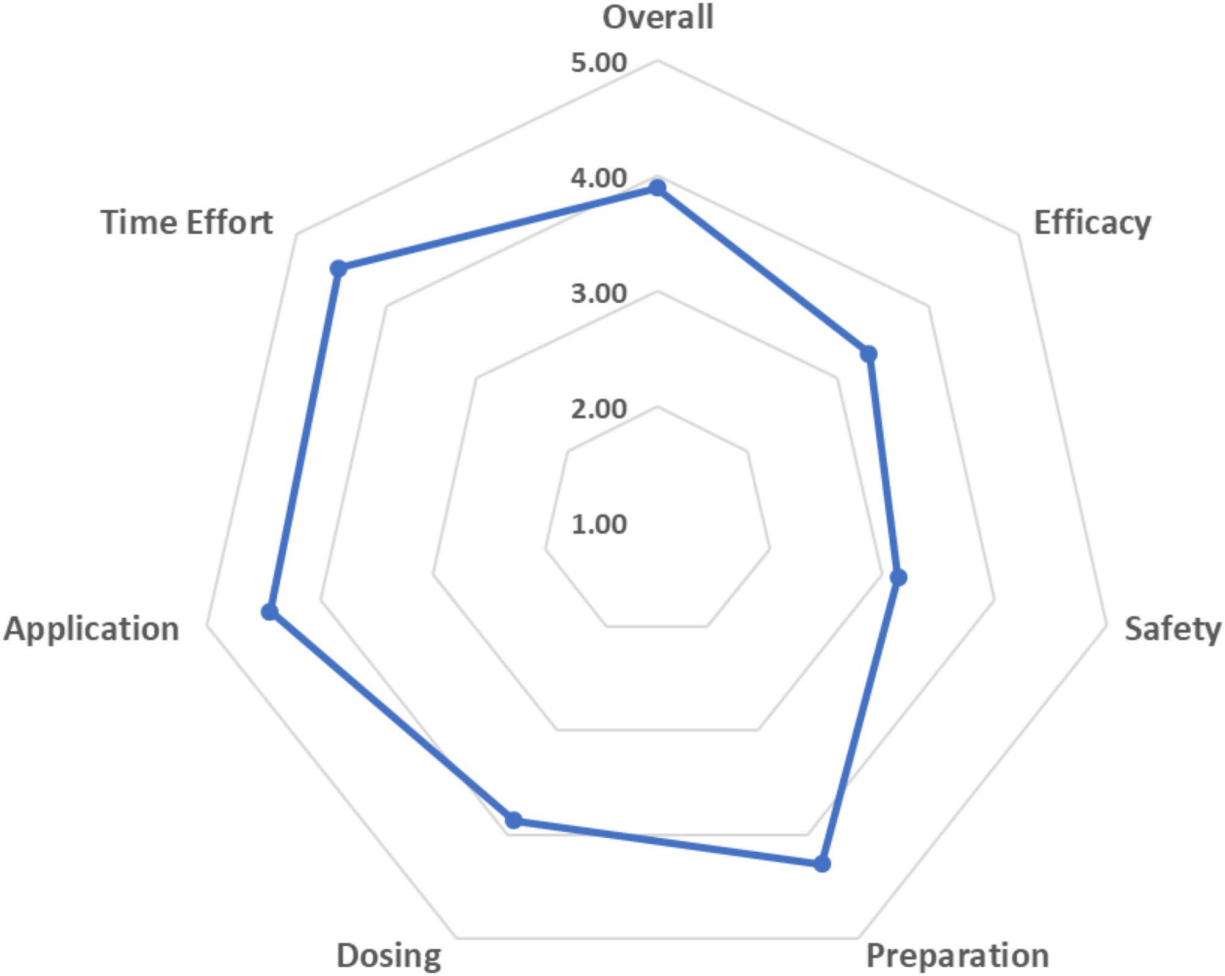
Spider Plot of the survey results. Ratings regarding TNK use are scaled as follows: 5 = much better, 4 = better, 3 = similar, 2 = worse, and 1 = much worse compared to rt-PA. Survey results were available from 40 health care providers (11 nurses, 29 physicians)

The survey, assessed 3 months after transition to TNK, revealed high satisfaction with TNK among local healthcare providers, responded by 73% physicians and 27% nursing staff (Fig. 3 and Supplementary Table 1). Clinical work experience varied, with the majority having over 10 years of experience (64%). TNK use was frequent, with 70% of respondents reporting repeated use and 17% regular use. Most participants (68%) rated TNK as better or much better than rt-PA. Drug preparation, dosing, and application were rated superior by 86%, 67%, and 97%, respectively. With 94% noting time efficiency improvements. Notably, the subjective median DTN time saving was estimated at 5 min. Preferences favored TNK (42%) or expressed neutrality (47%), with only 11% preferring rt-PA.

## Discussion

This real-world study of 276 consecutive thrombolyzed patients demonstrates that transitioning from rt-PA to TNK as the standard thrombolytic agent in AIS management is safe, feasible and associated with meaningful benefits in a tertiary care setting. Several aspects deserve attention.

Our findings highlight a significant reduction in actual DTN times by a median of 7 min with TNK. In AIS with LVO, an additional 7 min of ischemia time results in the loss of approximately 13 million more neurons, underscoring the clinical importance of streamlined thrombolysis workflows [23]. Previous studies have similarly reported shortened DTN and DTG times after transitioning to TNK, primarily due to its single-bolus administration and ease of preparation [4, 24–28]. A closer look at our treatment times shows, that the times from DTI between both groups did not differ significantly. However, a significant reduction in ITN time was observed with TNK, suggesting that the improvement in DTN times with TNK was primarily driven by the easier preparation and administration of TNK itself, leading to earlier treatment initiation after imaging diagnosis. Comparable findings were observed in an earlier study, where the faster DTN time with TNK was similarly driven by a significantly faster ITN time [26]. TNK’s simplified administration also accelerates preparation for EVT, which often involves anesthesia and intubation. Unlike rt-PA, TNK avoids the need for a second intravenous catheter for infusion, a logistical advantage in emergent settings [28].

While we observed a higher proportion of patients achieving excellent or good neurological recovery at discharge with TNK, statistical significance was not reached. Larger sample sizes, as achieved in meta-analyses, are likely needed to detect significant differences in functional outcomes [12]. Our findings align with existing evidence demonstrating comparable efficacy and safety between TNK and rt-PA in AIS [5–11]. TNK did not lead to significantly increased rates of hemorrhagic complications in our small cohort, even in high-risk groups such as those presenting beyond 4.5 h or under concurrent anticoagulation, but our study may be underpowered to detect small differences. Safety outcomes were reassuring without significant differences among TNK and rt-PA treated patients, overall. Somehow, a small numerical but non-significant increase of any ICH was observed in the subgroup analysis of patients with moderate strokes, (NIHSS 5–15) in which TNK treatment was associated with a numerically higher rate of ICH compared to rt-PA. But, upon further review, some cases were attributed to alternative causes, such as hemorrhagic brain metastases or procedural complications during EVT. This highlights the need for cautious interpretation of subgroup findings in this retrospective analysis of a relatively small-sized stroke cohort, further research is needed to assess potential confounding factors.

The successful implementation of TNK at our center underscores the importance of multidisciplinary education and comprehensive preparation. Our training program emphasized TNK’s pharmacology, dosing, and administration differences from rt-PA to mitigate the risk of dosing errors. Exclusive use of stroke-specific TNK vials (max. 25 mg), distinct from myocardial infarction doses, and full transition to TNK—including off-label scenarios—were key safeguards. Previous studies have similarly demonstrated the importance of staff training and standardized protocols in facilitating safe transitions [19, 20, 27]. Our survey revealed high satisfaction among healthcare providers, with clear advantages for TNK in preparation, dosing, administration, and time efficiency. Notably, TNK was favored by a majority of respondents or regarded as equivalent to rt-PA, reflecting its practical benefits in acute care workflows. The positive reception among staff supports TNK’s broader adoption in stroke centers.

This study has limitations that warrant consideration. First, it was conducted at a single center which may limit generalizability of our results to other hospitals with varying resources and protocols. However, similar improvements in time metrics have been reported in multicenter studies [25, 27]. Second, our subgroup analyses– particularly for patients treated beyond 4.5 h or under concurrent intake of anticoagulation– were limited by small sample sizes. Larger studies are needed to confirm our preliminary findings in these populations. Third, the retrospective design precludes randomization, leaving potential confounders unaccounted for. The two treatment groups were not perfectly matched, as there were slight imbalances in baseline characteristics. The alteplase group included a higher proportion of patients with cardiovascular comorbidities such as arterial hypertension, vascular diseases, and atrial fibrillation. Additionally, more patients in the alteplase group had large vessel occlusions in the carotid-T segment, which is associated with a worse prognosis. Furthermore, the proportion of patients receiving mechanical thrombectomy was slightly lower in the rt-PA group compared to the TNK group as was the proportion of patients with a premorbid mRS score of 0–1. While these differences may have affected clinical outcomes such as mRS at discharge, the procedural times as our primary endpoints should not have been influenced by it. Fourth, our analysis was limited to in-hospital outcomes, and longer follow-up is necessary to evaluate the sustained impact of TNK on functional recovery.

## Conclusions

Our findings support the complete transition to TNK for IVT in AIS. The increased simplicity of treatment, enhanced workflow efficiency, and high satisfaction among medical staff, combined with comparable functional and safety outcomes, establish TNK as a practical and effective thrombolytic agent in routine clinical practice.

## Electronic supplementary material

Below is the link to the electronic supplementary material.

Supplementary Material 1

Supplementary Material 2

## Data Availability

The data that support the findings of this study are not publicly available due to ethical restrictions but may be available from the corresponding author upon reasonable request and with appropriate permissions.

## Abbreviations

AIS: Acute ischemic stroke
CT: Computed tomography
DOAC: Direct oral anticoagulants
DTG: Door-to-groin
DTI: Door-to-imaging
DTN: Door-to-needle
DTR: Door-to-recanalization
ER: Emergency room
EVT: Endovascular treatment
ICH: Intracranial hemorrhage
INR: International normalized ratio
IQR: Interquartile range
ITN: Imaging-to-needle
IVT: Intravenous thrombolysis
LVO: Large vessel occlusion
MRI: Magnetic resonance imaging
mRS: Modified Rankin Scale
mTICI: Modified Thrombolysis in Cerebral Infarction
NIHSS: National Institutes of Health Stroke Scale
OAC: Oral anticoagulation
PH: Parenchymatous hematoma
rt-PA: recombinant tissue plasminogen activator or alteplase
SD: Standard deviation
sICH: Symptomatic intracranial hemorrhage
SOP: Standard operating procedure
TNK: Tenecteplase
