# Supplementary material for: Switch to tenecteplase for intravenous thrombolysis in stroke patients: experience from a German high-volume stroke center
Source: Neurol Res Pract. 2025 May 5;7(1):28. doi: 10.1186/s42466-025-00388-x (PMC12051303; doi:10.1186/s42466-025-00388-x)
Supplement: Supplementary file 2 — Supplementary Material 2 [file 42466_2025_388_MOESM2_ESM.docx]

**Supplementary Material**

*Sekita et al. Switch to Tenecteplase for intravenous thrombolysis in stroke patients: Experience from a German High-volume Stroke Center*

**Content:**

**sFigure 1: Median Procedure times comparing TNK vs. rt-PA in**

**(A) any AIS and**

**(B) AIS within a time window of 4.5 hours and NIHSS>4 on presentation.**

**sFigure 2: Bleeding events comparing TNK vs. rt-PA treated patients.**

**sTable 1: Survey results on TNK perceptions among health care providers**


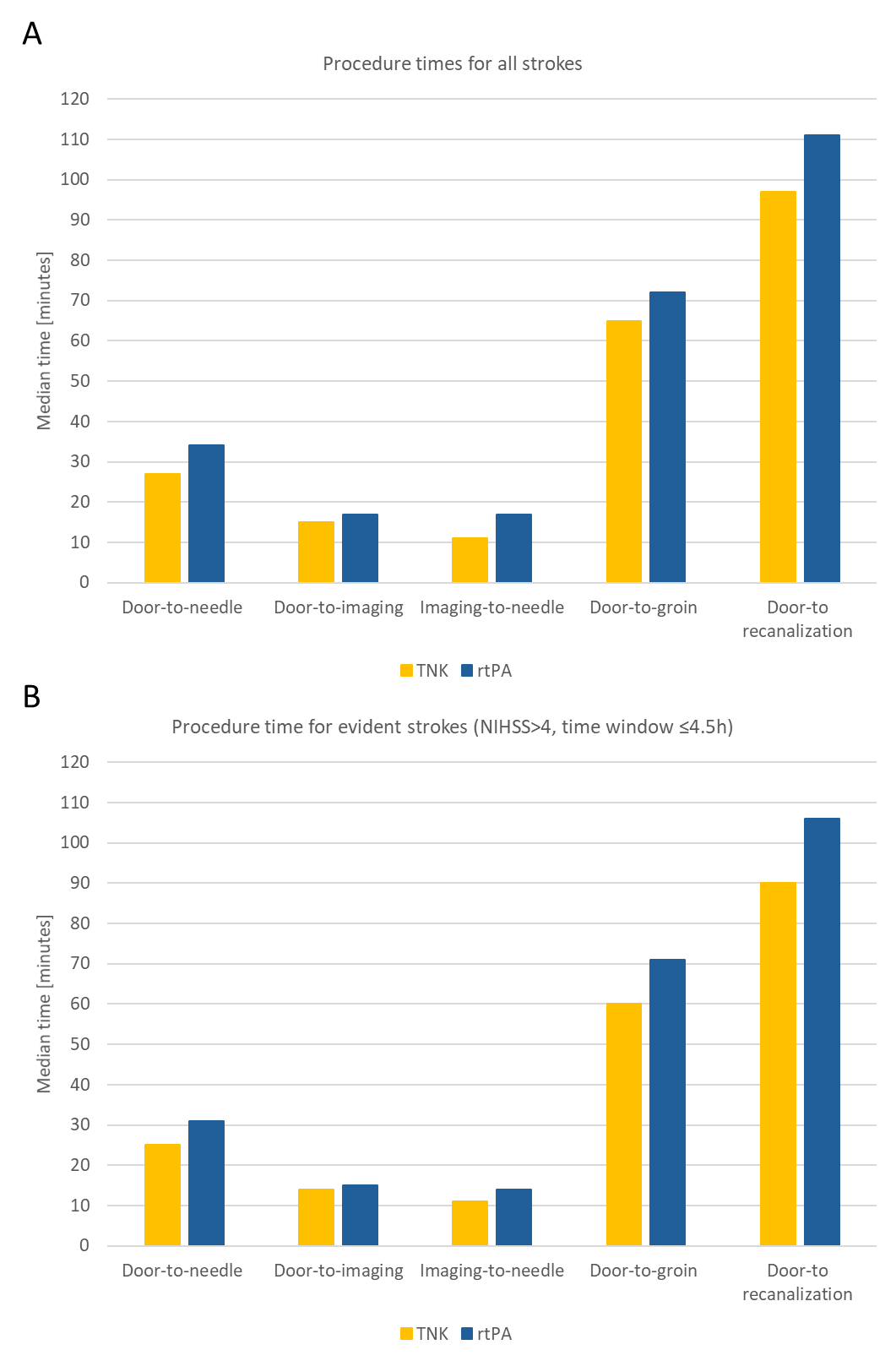


**Supplementary Figure 1: Median procedure times comparing TNK vs. rt-PA in (A) any AIS and (B) AIS within a time window of 4.5-hours and NIHSS>4 on presentation.** Abbreviations: NIHSS, National Institutes of Health Stroke Scale


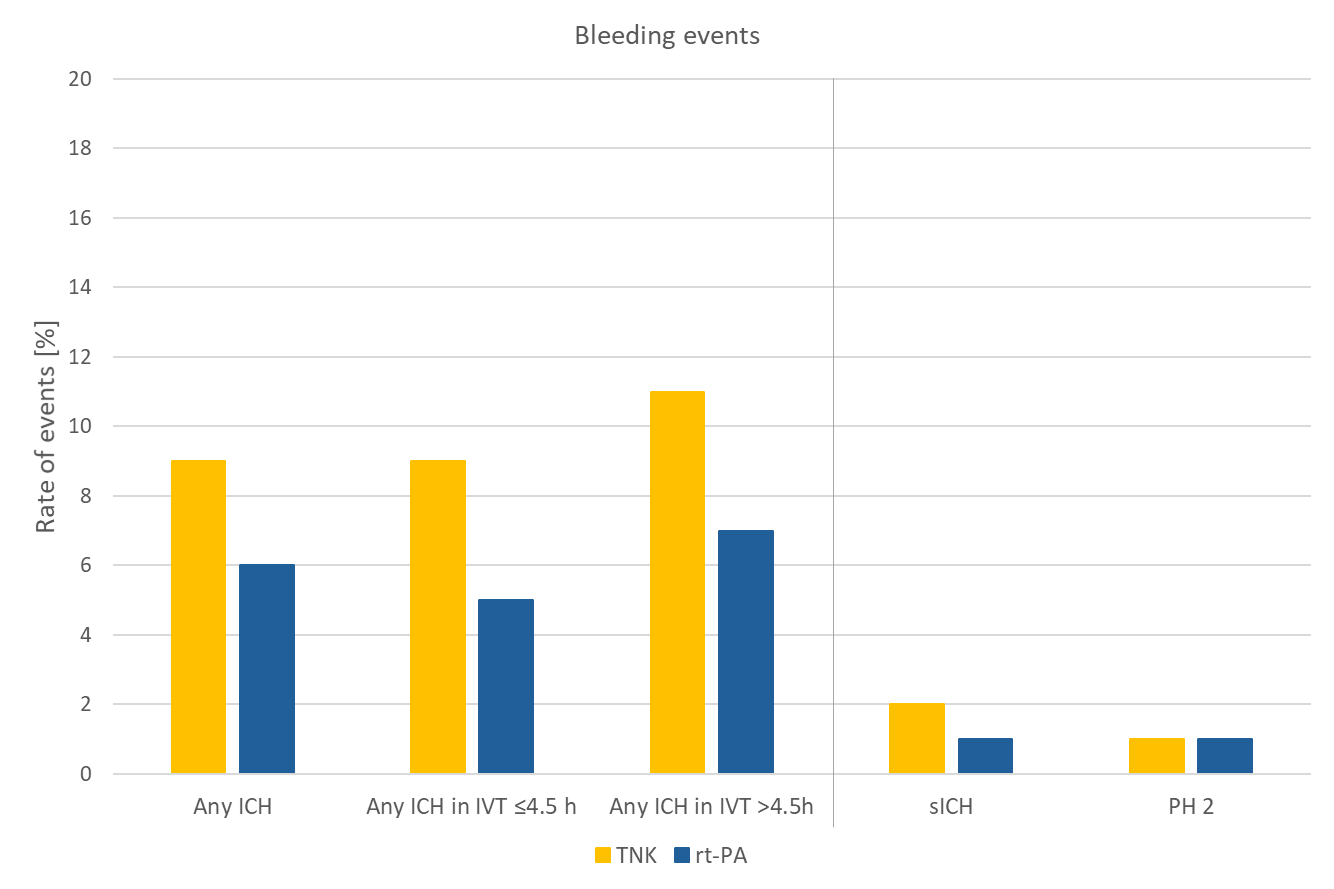


**Supplementary Figure 2: Bleeding events comparing TNK vs. rt-PA treated patients.** Bleeding events were categorized into any ICH, symptomatic ICH according to SITS-MOST criteria, and parenchymatous hematoma (PH2; Heidelberg Bleeding Classification). Further subanalysis was performed for IVT performed within 4.5-hours and beyond 4.5-hours time from symptom onset. Abbreviations: ICH indicates intracranial hemorrhage; IVT, intravenous thrombolysis; sICH, symptomatic intracranial hemorrhage; PH, parenchymatous hematoma.

**Supplementary Table 1: Survey results on TNK perceptions among health care providers**

| **Question** | **Response Options** | **Results** |
| --- | --- | --- |
| Professional Group; n (%) | Physician  Nursing staff | 29 (73%)  11 (27%) |
| Clinical experience of physicians; n (%) | Assistant doctor (IMC/ICU)  Assistant doctor (post-acute care unit)  Specialist doctor  Senior physician | 13 (46%)  5 (18%)  4 (14%)  6 (21%) |
| Lysis behavior changed after transition; n (%) | No  Yes | 10 (100%)  0 (0%) |
| Level of education for nursing staff; n (%) | Apprentice  Health care professional  Specialized training ICU  Specialized training IMC | 0 (0%)  6 (60%)  0 (0%)  4 (40%) |
| Clinical work experience, years; n (%) | <3  3-6  7-10  ≥10 | 0 (0%)  3 (27%)  1 (9%)  7 (64%) |
| Frequency of TNK use; n (%) | Never  Once  Repeatedly  Regularly | 3 (8%)  2 (6%)  25 (70%)  6 (17%) |
| **Assessment of following aspects; n (%)** |  |  |
| Overall satisfaction | Much worse than rt-PA  Worse than rt-PA  Like rt-PA  Better than rt-PA  Much better than rt-PA | 0 (0%)  1 (3%)  9 (29%)  13 (42%)  8 (26%) |
| Efficacy | Much worse than rt-PA  Worse than rt-PA  Like rt-PA  Better than rt-PA  Much better than rt-PA | 0 (0%)  0 (0%)  24 (71%)  8 (24%)  2 (6%) |
| Safety | Much worse than rt-PA  Worse than rt-PA  Like rt-PA  Better than rt-PA  Much better than rt-PA | 0 (0%)  8 (22%)  19 (53%)  5 (14%)  4 (11%) |
| Drug preparation | Much worse than rt-PA  Worse than rt-PA  Like rt-PA  Better than rt-PA  Much better than rt-PA | 0 (0%)  0 (0%)  5 (14%)  16 (44%)  15 (42%) |
| Dosing | Much worse than rt-PA  Worse than rt-PA  Like rt-PA  Better than rt-PA  Much better than rt-PA | 0 (0%)  2 (6%)  10 (28%)  15 (42%)  9 (25%) |
| Application | Much worse than rt-PA  Worse than rt-PA  Like rt-PA  Better than rt-PA  Much better than rt-PA | 0 (0%)  0 (0%)  1 (3%)  18 (50%)  17 (47%) |
| Time expenditure | Much worse than rt-PA  Worse than rt-PA  Like rt-PA  Better than rt-PA  Much better than rt-PA | 0 (0%)  0 (0%)  2 (6%)  13 (36%)  21 (58%) |
| Estimated time saving in DTN by TNK, min; mean |  | 5 |
| Preference of thrombolytic therapy; n (%) | TNK  rt-PA  No preference | 15 (42%)  4 (1%)  17 (47%) |

Abbreviations: IMC, intermediate care; ICU, intensive care unit
