# Supplementary material for: Switch to tenecteplase for intravenous thrombolysis in stroke patients: experience from a German high-volume stroke center
Source: Neurol Res Pract. 2025 May 5;7(1):28. doi: 10.1186/s42466-025-00388-x (PMC12051303; doi:10.1186/s42466-025-00388-x)
Supplement: Supplementary file 1 — Supplementary Material 1 [file 42466_2025_388_MOESM1_ESM.docx]

**Response to reviewers**

**Manuscript:**	Switch to Tenecteplase for intravenous thrombolysis in stroke patients: Experience from a German High-volume Stroke Center

**Editor:**

*Authors are requested to consider widening of the time period (+/- 6 months before and after switching to tenecteplase) for analysis to improve significance and meaningfulness of this interesting study.*

Response to the editor:

We thank the editor for this helpful suggestion. We managed to widen our time-period to 6 months before and after TNK-transmission and are now confident that our completely new analyses, based on around 150 AIS-patients per thrombolytic agent, are more significant and robust. We have updated the manuscript accordingly and revised all relevant sections (underdone changes highlighted in yellow). We are confident that our revised manuscript is now suitable for publication in Neurological Research and Practice.

Please find below the detailed point-to-point response to all raised comments by the reviewers.

.

**Reviewer I**

*This is an interesting and timely manuscript regarding the use of tenecteplase (TNK) in acute stroke. It adds valuable real-world information to the existing literature by showing shorter door to needle times compared to rt-PA and substantial practical advantages. In addition, the save use in the extended time window is of interest.*

Response to the Reviewer:

We would like to thank the reviewer for his constructive feedback on our manuscript Your comments are greatly appreciated, and we thank the reviewer for the overall positive feedback.

*Comment I:*

*I have only one minor suggestion for change:*

*In the abstract, the conclusion should be even more cautious regarding safety.*

*In the study, any ICH and sICH was indeed more frequent with TNK, both in the <4.5 and the 4.5h window, although not statistically significant. It cannot be ruled out that the lack of significance was due to the small sample size. This is well discussed in the manuscript, but the quick reader focusing on the conclusion may get the impression that safety was no issue at all.*

Response to the Reviewer:

We thank the reviewer and totally agree that due to the initial limited sample size, interpretation of the results should be discussed very cautiously. We have now adapted the corresponding section in the abstract, accordingly. Further, after extension of the study period to ±6 months, we have doubled the sample size and still did not observe significant changes among TNK- and rt-PA treated patients.

**Reviewer II:**

*The authors report on the single center experience with the transition from Alteplase (rt-PA) to Tenecteplase (TNK) for intravenous thrombolysis (IVT) in acute ischemic stroke (AIS). In retrospective analysis they compared process parameters as well as treatment and safety outcomes for AIS patients treated with rt-PA before the transition and those treated with TNK afterward over a period of three months before and three months after transition to TNK. Results showed significantly shorter door-to-needle (DTN) times with TNK safety outcomes, including rates of intracranial hemorrhage and angioedema, remained comparable (although hemorrhage rates were numerically higher with TNK). Medical staff reported high satisfaction with TNK, particularly regarding its ease of preparation and administration. Taken together, these findings suggest that implementing TNK in routine stroke care is feasible, improves process parameters, and maintains safety. The study supports TNK's advantages as a practical alternative to rt-PA in acute stroke management.*

*The paper is well written, background, objectives, and methods are clear. Results are presented in a clear fashion, and the discussion is based on the data and covers most important aspects. I have only one major comment:*

*Response to the Reviewer:*

We thank the reviewer for the overall positive evaluation and constructive feedback, please find below the point-to-point response to the raised issues.

*Reviewer’s comment 1:*

*The time period studied (3 months each) is really short and so the numbers of patients are small and results are susceptible to chance and possible seasonal influence. If an extension of the time period analyzed to 6 months would be possible, this would stingray improve the quality of the paper.*

Response to the reviewer:

We thank again the reviewer and the editor for this helpful comment. As mentioned above, we have extended the time window of the study to ±6 months to improve the sample size and reduce potential biases related to the limited sample size. We have also included the imaging-to-needle time in our analysis to rule out potential confounding. We are confident that our revised manuscript - based on completely new analyses based on 150 -AIS patients per thrombolytic agent – is no suitable for publication in NRP.

*Reviewer’s comment 2:*

*In the discussion, the numerically higher bleeding rates for TNK might be mentioned (while I would not discuss p-values as significant from multiple uncontrolled testing, i.e. the one single "significant" difference in bleeding rates ("except for higher ICH rates in TNK-treated patients with moderate strokes (NIHSS 5-15; TNK 14% vs. rt-PA 0%, p=0.036)")*

*Response to the reviewer:*

We thank the reviewer for this valueable comment. In our new analysis – based on 138 TNK treated and 138 rt-PA treated patients, we observed a rate of any ICH in 8/62 (13%) TNK and 2/68 (3%) in rt-PA treated patients with moderate stroke (i.e. on admission NIHSS 5-15; p=0.06). However, as this trend was only observed in one subgroup and only for any ICH, it may be due to chance and due to multiple testing performed for our subgroup-analyses. However, to notice the reader about this observation, we have now added a corresponding section in the discussion and expanded our limitation section, accordingly.

*Reviewer’s comment 3:*

*Hemorrhage rates should be presented in a table in the main text, not as supplement.*

Response to the reviewer:

We appreciate this helpful comment and created a revised Table 3. Further, we have put the corresponding figure into the supplementary material, accordingly (new supplemental Figure 2).

*Reviewer’s comment 4:*

*Table 1: p-Values for baseline demographics are unusual and not necessary*

Response to the reviewer:

We appreciate this valueable comment and adapted our Table 1, according to the reviewer’s suggestion.

*Reviewer’s comment 5:*

*Table 1: TOAST does not include ESUS, bot unknown etiology (not necessarily ESUS)*

Response to the reviewer:

We agree with the reviewer and are now more precise in the revised Table 1.

*Reviewer’s comment 6:*

*Table 2: units for times should be given (minutes)*

Response to the reviewer:

The units for procedure times are provided in the section heading (see Table 2 – procedure times, min). We hope that this approach is in line with the reviewer.
